# Somatic genetic variation shapes clonal selection of memory NK cells

**DOI:** 10.64898/2026.09.17.752421

**Published:** 2026-11-03

**Authors:** Timo Rückert, Matthias Günther, Oliver Knight, Maximilian Mandry, Karl Junker, Simon Grassmann, Joseph C. Sun, Thomas Höfer, Chiara Romagnani

## Abstract

In adaptive immunity, antigen receptor specificity and affinity drive clonal selection, but what enables individual innate lymphocytes to gain a selective advantage without rearranged antigen receptors remains unknown. We traced somatic mutations in human natural killer (NK) cells, revealing extensive clonal dominance in cytomegalovirus-associated memory, with individual genetically diversified clones comprising up to 95% of the population, originating early in life and expanding over years. Their genetic evolution was associated with positive selection of acquired coding mutations, linking somatic variation to NK cell clonal fitness. Among candidate drivers, we identified mutations in the chromatin regulator KMT2D, whose induced deletion in mature NK cells conferred a competitive advantage following infection in vivo. These findings establish somatic evolution as a fundamental mechanism shaping innate immune memory.

